# Rapid and efficient production of cecropin A antibacterial peptide in *Escherichia coli* by fusion with a self-aggregating protein

**DOI:** 10.1186/s12896-018-0473-7

**Published:** 2018-10-05

**Authors:** Meng Wang, Kaiwen Zheng, Jinglian Lin, Minhua Huang, Yi Ma, Shan Li, Xiaochun Luo, Jufang Wang

**Affiliations:** 1 School of Biology and Biological Engineering, South China University of Technology, Guangzhou, 510006 China; 2 Guangdong Key Laboratory of Fermentation and Enzyme Engineering, School of Biology and Biological Engineering, South China University of Technology, Guangzhou, 510006 China

**Keywords:** Self-aggregating protein, Cecropin A, Antimicrobial peptide, ELK16, *Escherichia coli*

## Abstract

**Background:**

Cecropin A (CeA), a natural cationic antimicrobial peptide, exerts potent antimicrobial activity against a broad spectrum of Gram-positive and Gram-negative bacteria, making it an attractive candidate substitute for antimicrobials. However, the low production rate and cumbersome, expensive processes required for both its recombinant and chemical synthesis have seriously hindered the exploitation and application of CeA. Here, we utilized a short β-structured self-aggregating protein, ELK16, as a fusion partner of CeA, which allowed the efficient production of high-purity CeA antibacterial peptide with a simple inexpensive process.

**Results:**

In this study, three different approaches to the production of CeA peptide were investigated: an affinity tag (His-tag)-fused protein expression system (AT-HIS system), a cell-free protein expression system (CF system), and a self-assembling peptide (ELK16)-fused protein expression system (SA-ELK16 system). In the AT-HIS and CF systems, the CeA peptide was obtained with purities of 92.1% and 90.4%, respectively, using one or more affinity-chromatographic purification steps. The procedures were tedious and costly, with CeA yields of only 0.41 and 0.93 μg/mg wet cell weight, respectively. Surprisingly, in the SA-ELK16 system, about 6.2 μg/mg wet cell weight of high-purity (approximately 99.8%) CeA peptide was obtained with a simple low-cost process including steps such as centrifugation and acetic acid treatment. An antimicrobial test showed that the high-purity CeA produced in this study had the same antimicrobial activity as synthetic CeA peptide.

**Conclusions:**

In this study, we designed a suitable expression system (SA-ELK16 system) for the production of the antibacterial peptide CeA and compared it with two other protein expression systems. A high yield of high-purity CeA peptide was obtained with the SA-ELK16 system, which greatly reduced the cost and time required for downstream processing. This system may provide a platform for the laboratory scale production of the CeA antibacterial peptide.

**Electronic supplementary material:**

The online version of this article (10.1186/s12896-018-0473-7) contains supplementary material, which is available to authorized users.

## Background

Antimicrobial peptides (AMPs) are small peptides of 10–50 amino acids that are effective in the innate immune systems of a wide variety of organisms, including bacteria, plants, insects, and mammals [1–3]. In general, they are cationic, amphipathic, β-folded or α-helix peptides that display rapid, strong, and broad-spectrum activities against a wide range of pathogens, including bacteria, fungi, plants, and even insects [4–6].

In recent decades, the widespread use of antimicrobials has resulted in a rapid increase in antimicrobial resistance, which has increased the worldwide morbidity [7] and economic losses [8, 9]. This has led to enormous efforts to explore and develop new antimicrobial agents, such as AMPs. Moreover, because of their broad spectrum of antibacterial properties but no hemolytic or cytotoxic activities, AMPs are considered attractive targets for the development of new antimicrobial compounds [10, 11]. Cecropin A (CeA) is a natural linear cationic α-helical AMP isolated from insects [12–14]. It contains a strongly cationic region at its N-terminus and a large hydrophobic tail at its C-terminus, which allow its interaction with the microbial membrane, and then induces cell lysis by pore formation [1]. It has a wide spectrum of antimicrobial activities against Gram-negative bacteria, Gram-positive bacteria, and fungal phytopathogens [15]. More importantly, this peptide does not induce the lysis of erythrocytes or lymphocytes, even at high concentrations [16]. Therefore, the CeA peptide has become a potentially useful antimicrobial substance in biochemical and pharmaceutical research [17].

Because the chemical synthesis of the CeA peptide or extraction from natural sources are extremely expensive, with low yields [6, 18–20], the development of a simple way to efficiently produce high-purity CeA peptide is urgently required. In recent years, there has been much research into the production of CeA and its derivatives [21–24]. To reduce the production costs, *Pichia pastoris* and *Bacillus subtilis* have been used as the expression hosts for the secretion of the CeA peptide [23, 25, 26]. To avoid the formation of inclusion bodies, some researchers have fused the peptide to glutathione S-transferase (GST) or thioredoxin (TRX), which significantly increased the soluble fraction of the peptide [16, 21, 27]. To obtain high-purity CeA peptide, affinity tags such as the hexahistidine (His_6_) tag, cysteine protease domain tag, and strep tag, have been used, and high-purity peptide produced [24, 28, 29]. However, all these methods still require expensive purification with affinity chromatography or high-performance liquid chromatography and the yields are relatively low. More importantly, the peptide toxicity for host cells and the proteolysis of the product are still two obstacles to the high production of the CeA peptide.

Recently, a small β-strand self-assembling peptide, ELK16, which induces the formation of “active inclusion bodies” in vivo, has attracted the attention of many researchers [30, 31]. Based on the self-assembling property of ELK16, a CeA fusion protein containing *Mxe* GyrA intein and ELK16 was constructed and expressed in *E. coli* BL21(DE3) cells (SA-ELK16 system). Besides, two other approaches were also investigated: an affinity tag (His tag) fusion protein expression system (AT-HIS system) and a cell-free protein expression system (CF system). We compared the costs and yields of each process, and thus established a simple, inexpensive, single-step purification method for the efficient production of high-purity CeA peptide. This method provides a novel platform for the laboratory large-scale production of CeA peptide.

## Results

### Design and construction of three different CeA fusions

**Fig. 1:**
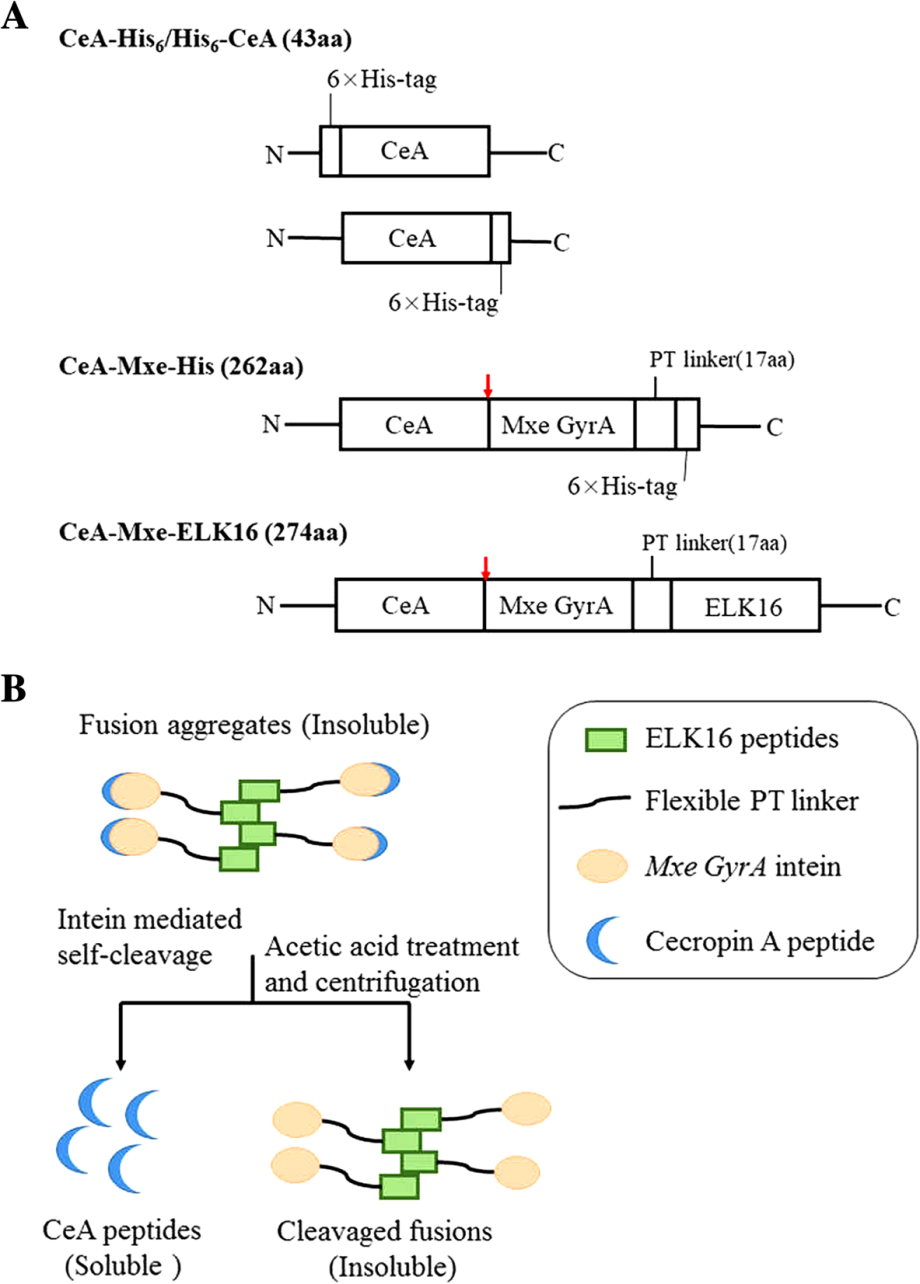
Schematic diagram of three different fusions and quick purification of CeA–Mxe–ELK16 fusion. **a** Compositions of the three different fusions (CeA–Mxe–His, CeA–His_6_ or His_6_–CeA, and CeA–Mxe–ELK16) used in the three different expression systems (AT-HIS system, CF system, and SA-ELK16 system, respectively). “N” and “C” represent the amino and carboxy terminals of the polypeptide; Red arrow indicates the cleavage site of *Mxe* GyrA intein. **b** Easy simple purification process for CeA–Mxe–ELK16 fusion

Because AMPs are toxic to host cells and are readily degraded by proteases [32], various proteins such as small ubiquitin-related modifier (SUMO) [33, 34], GST [35], and TRX [31, 36] were selected as fusion partners for the efficient expression of AMPs. Here, we incorporated a self-cleaving peptide *Mxe* GyrA intein [37] as the fusion carrier in the AT-HIS system to avoid the degradation of CeA by proteases to improve its expression. High-purity CeA peptide was released by the self-cleavage of *Mxe* GyrA intein and then purified with nickel ion affinity chromatography (fusion mode “CeA–Mxe–His”, shown in Fig. 1a). In the CF expression system, a His_6_ tag was fused to the CeA peptide by RF cloning to allow its downstream purification. RF cloning is a simple and restriction-free method for insertion of foreign DNA into a plasmid at any desired location [38]. To achieve the high-level expression of the CeA peptide, two different fusions were constructed: N-terminal fusion (His_6_–CeA) or C-terminal fusion (CeA–His_6_) (fusion mode “His_6_–CeA” or “CeA–His_6_”, respectively; shown in Fig. 1a). Recent studies have reported that some peptides like ELK16 can assemble into aggregates when expressed in *E. coli*. Based on the results of this study, ELK16 was selected and attached to the C-terminus of *Mxe* GyrA intein (CeA–Mxe–ELK16) in the SA-ELK16 system to induce and increase the formation of active fusion protein aggregates in *E. coli* (fusion mode “CeA–Mxe–ELK16”, shown in Fig. 1a). These aggregates were then separated by simple centrifugation and high-purity CeA peptide was successfully released into soluble fractions after dithiothreitol (DTT)-induced *Mxe* GyrA intein cleavage and acetic acid treatment (Fig. 1b).

### Expression, optimization and purification of CeA peptide with the AT-HIS system

**Fig. 2:**
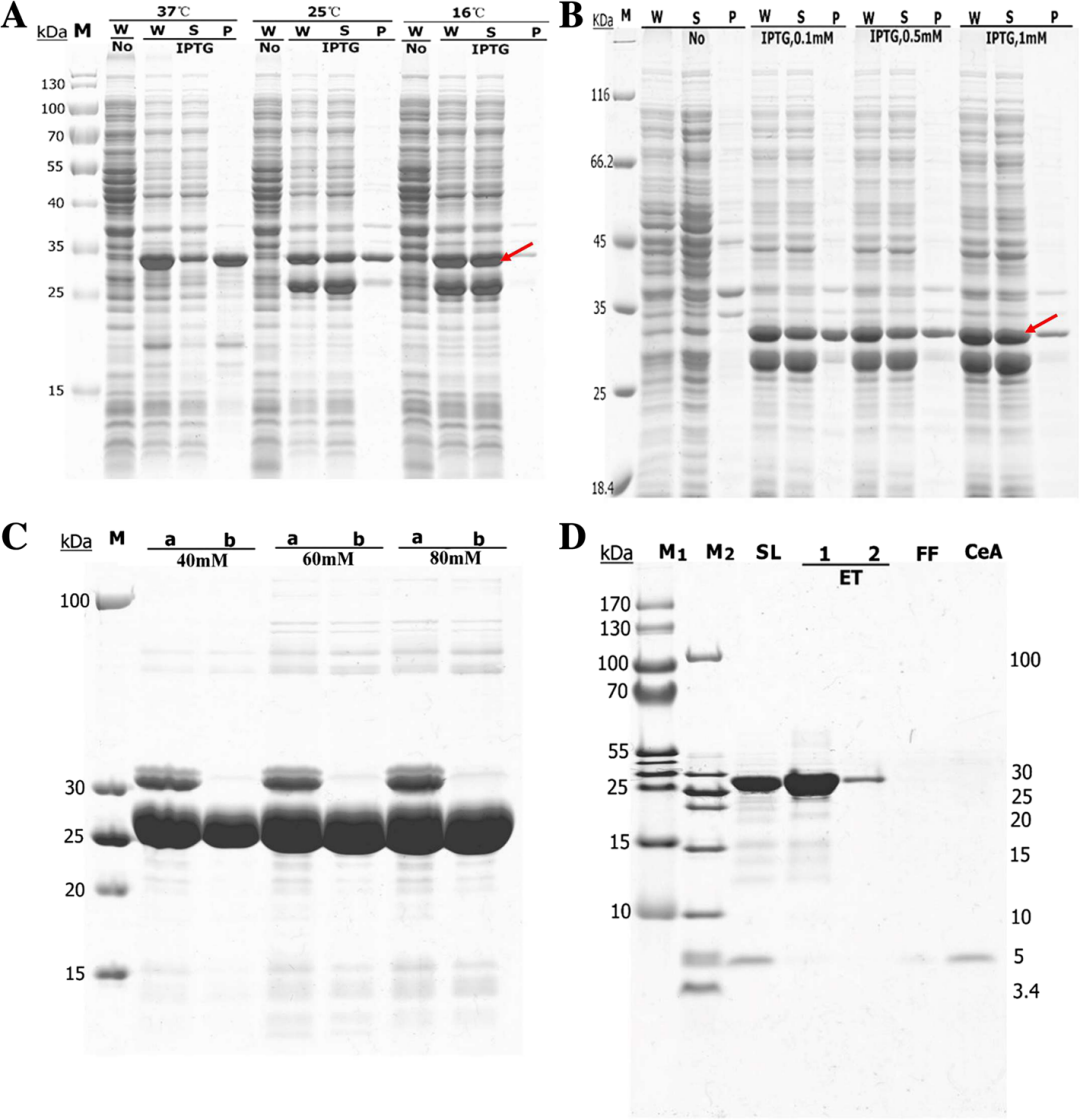
Expression, optimization, and purification of CeA peptide with AT-HIS system in *E. coli*. Competent *E. coli* BL21(DE3) cells were transformed with plasmid pET21–CeA–Mxe–His, which produced the CeA–Mxe–His fusion. Cells carrying plasmid pET21–CeA–Mxe–His were cultured in LB medium to express the fusion. To induce high expression, different temperatures (16 °C, 25 °C, or 37 °C) (**A**) and a series of IPTG concentrations (**B**) were used. Different concentrations of DTT (40–80 mM) were tested to optimize the efficiency of *Mxe* GyrA intein cleavage (**C**). High-purity CeA peptide was obtained after nickel ion affinity chromatography (**D**). Red arrow indicates CeA–Mxe–His fusion; NO, the cells were not induced with IPTG; W, whole-cell lysate; S, supernatant fractions; and P, insoluble pellets of induced cells after sonication. “a” and “b” in C indicate the sample before and after cleavage by DTT, respectively. In D: SL, supernatant before it was loaded onto the column; ET, elution fraction; FF, fast-flow fraction; CeA, final concentrated CeA peptide. M, M_1_ and M_2_, protein markers of different molecular weights

*E. coli* BL21(DE3) cells harboring plasmid pET21a–AM–His were cultured at 37 °C and isopropyl β-D-1-thiogalactopyranoside (IPTG) was added when the optical density of the cells at a wavelength of 600 nm (OD600) reached 0.6–0.8 to initiate the expression of CeA–Mxe–His fusion. To achieve high-level expression of the soluble CeA–Mxe–His fusion, different incubation temperatures (37 °C, 25 °C, and 16 °C) were investigated. As shown in Fig. 2a, large amounts of the CeA–Mxe–His fusion were successfully expressed in *E. coli* BL21(DE3) cells at all temperatures (bands marked with red arrow). However, the fusion polypeptide was expressed and deposited primarily in inclusion bodies when the cells were incubated at 37 °C for 5 h. When the temperature was reduced to 25 °C or 16 °C, large amounts of soluble CeA–Mxe–His fusion were produced (Fig. 2a). Different concentrations of IPTG (0.1, 0.5, or 1 mM) were also tested. As shown in Fig. 2b, a sufficient amount of soluble fusion was produced after induction with 0.1 mM IPTG and there was no obvious increase in the amount of CeA–Mxe–His fusion when 0.5 or 1 mM IPTG was used. In summary, we selected 25 °C, 0.1 mM IPTG and 12 h as the optimal conditions for the expression of CeA–Mxe–His fusion.

The CeA–Mxe–His fusion was purified with nickel ion affinity chromatography and high-purity CeA–Mxe–His fusion was collected (Additional file 1). To obtain more CeA peptide, the efficiency of *Mxe* GyrA intein cleavage was optimized. Figure 2c shows that nearly 100% of the CeA–Mxe–His fusion was successfully cleaved by the addition of 40 mM DTT, which is consistent with other reports [34, 37], and the CeA peptide was successfully released from the fusion (the band at 5 kDa in line SL in Fig. 2d). Approximately 92.1% of the CeA peptide was obtained after a second round of nickel ion affinity chromatography, with a final yield estimated to be 0.41 μg/mg wet cell weight (Fig. 2d and Table 1). Unfortunately, severe intracellular self-cleavage of the CeA–Mxe–His fusion in the AT-HIS system could not be prevented, even when the low temperature (16 °C) and low concentration of IPTG (0.1 mM) were used, which greatly reduced the production of CeA peptide (two major bands in Fig. 2a and b).

**Table 1:** Summary of the expression and purification schemes in the three different systems

Expression systems	AT-HIS expression system	CF expression system	SA-ELK16 expression system
Required steps	Step 1: Expression of fusion protein CeA-Mxe-PT-His_6_	Step 1: Expression of antibacterial peptide His_6_-CeA	Step 1: Expression of fusion protein CeA-Mxe-ELK16
	Step 2: Cell disruption and supernatant collection	Step 2: Ni^2+^ affinity chromatography	Step 2: Cell disruption and acquisition of protein aggregates
	Step 3: Ni^2+^ affinity chromatography		Step 3: Intein-mediated cleavage
	Step 4: Intein-mediated cleavage		Step 4: acetic acid treatment and collection of CeA peptide.
	Step 5: The second Ni^2+^ affinity chromatography		
Time consuming	5 days	2 days	2 days
Final yield of peptide production (μg/mg wet cell pellet) ^a^	0.41	0.93 ^b^	6.20
Purity of bio-produced Cecropin A peptide (%)	92.1	90.4	99.8

^a^Yield of cecropin A peptide after intein-mediated cleavage in LB culture: 2.87 ± 0.62 mg/ml wet cell weight in the SA-ELK16 system and 1.48 ± 0.48 mg/ml wet cell weight in the AT-HIS system, estimated with the densitometric analysis software Gel-Pro Analyzer or BCA Protein Assay Kit

^b^Yield of CeA peptide in CF system, 0.93 μg/ml of reaction mixture

### Expression and purification of CeA peptide with the CF system

**Fig. 3:**
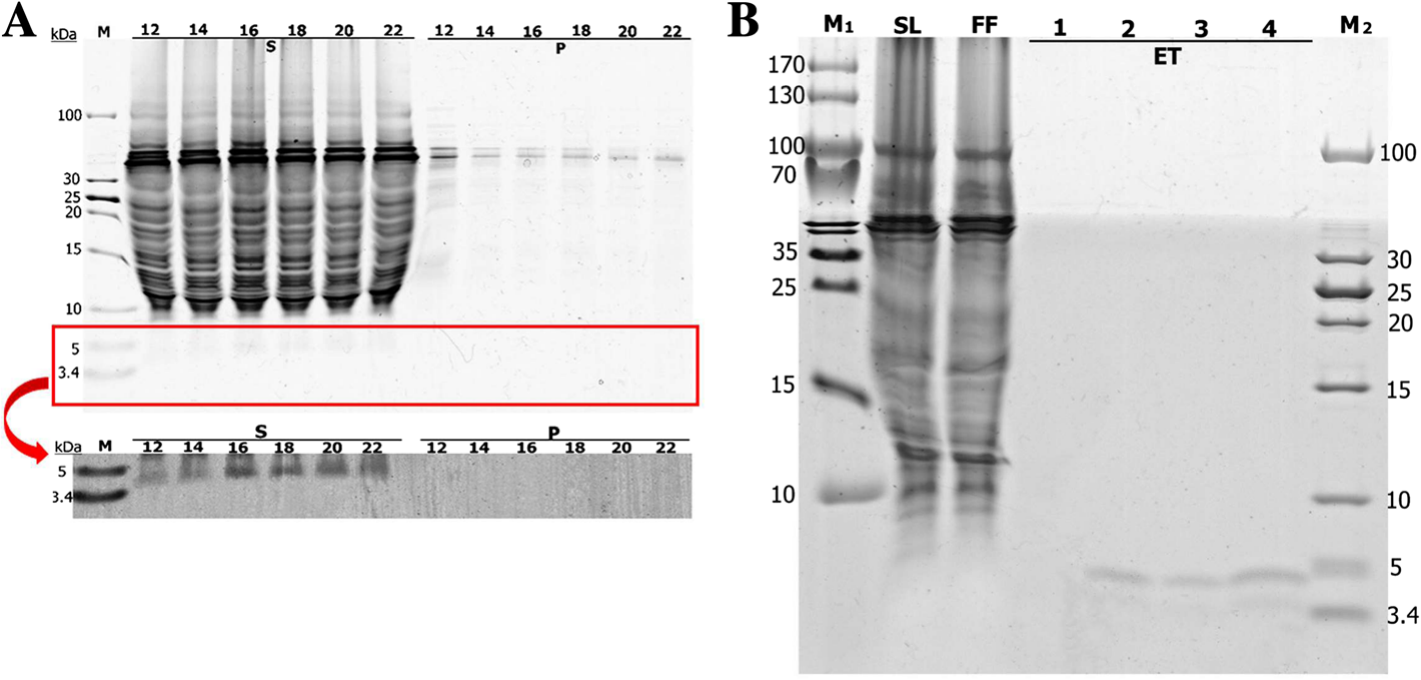
Expression and purification of CeA peptide in CF system. **a** Different Mg^2+^ ion concentrations (12–22 mM) were tested to optimize His_6_–CeA production in the CF system. The supernatant and pellet fractions of RM were separated by centrifugation at 12,000 g for 5 min and analyzed with tricine SDS–PAGE stained with Coomassie Blue. S, supernatant samples; P, pellet samples. Red frame contains the band of CeA peptide, processed with high gray-scale resolution. **b** Purification of His_6_–CeA with Ni^2+^-NTA affinity chromatography. SL, samples containing His_6_–CeA before purification; FF, flow-through fraction; ET (1–4), elution fractions with imidazole; M_1_, M_2_, different protein markers, in kDa

In the CF system, two different plasmids (pET21–His_6_–CeA and pET21–CeA–His_6_) were constructed to express the CeA peptide. The purified plasmid DNA was added as the template to the cell free reaction mixtures (RMs) and expressed at 30 °C for 16–18 h and then analyzed by tricine SDS-PAGE. However, the CeA peptide was not expressed when plasmid pET21–CeA–His_6_ was added to RM as the template (data not show). Fortunately, as shown in Fig. 3a, His_6_–CeA was successfully expressed as the soluble form, which suggests that the N-terminal fusion of the His tag improved the expression of the CeA peptide. Different concentrations (12–22 mM) of Mg^2+^ were also evaluated for enhanced expression of CeA peptide. Results in Fig. 3a shows that the expression of the CeA peptide increased with increments in the Mg^2+^ concentration and the highest production of soluble CeA peptide was achieved at an Mg^2+^ concentration of 16 mM. A further increase in the Mg^2+^ concentration did not improve the expression of the soluble CeA peptide, so an optimum Mg^2+^ concentration of 16 mM was selected.

The CeA peptide was purified with nickel ion affinity chromatography, as reported previously [39]. As shown in Fig. 3b, the amount of CeA peptide successfully purified was about 0.93 μg/ml of reaction mixture, with a purity of approximately 90.4% (Table 1).

### High-purity CeA peptide released in the SA-ELK16 system

**Fig. 4:**
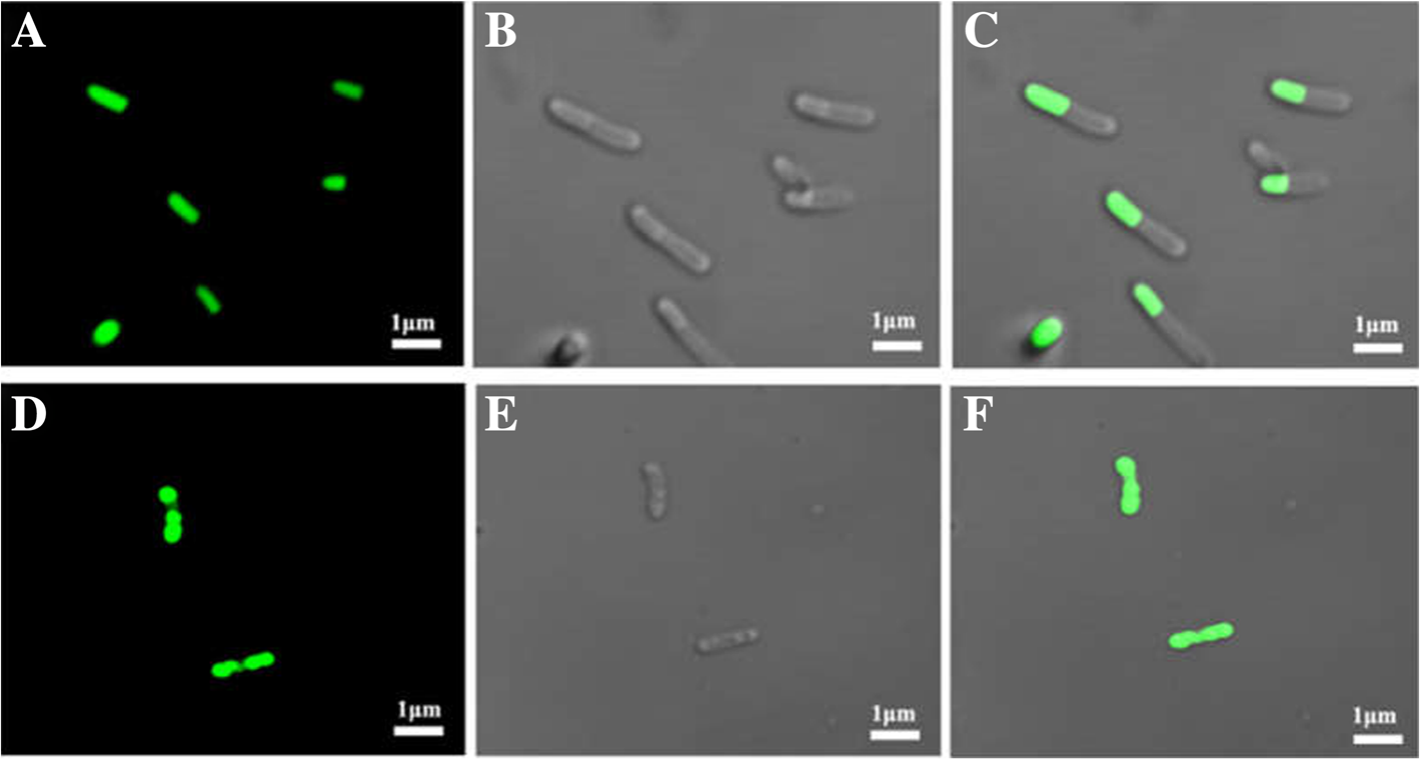
Differences in localization and form of the ELK16 fusion and HIS fusion in *E. coli*. **a**–**c** Confocal fluorescence micrographs of GFP–Mxe–ELK16 fusion protein; **d**–**f**, confocal fluorescence micrographs of GFP–Mxe–HIS fusion protein. **a** and **d** Green fluorescent images; **b** and **e**, bright-field images; **c** and **f**, merged images. Scale bars represent 1 μm

Previous studies have reported that some polypeptides like ELP, 18A, and ELK16 can induce the formation of aggregates in vivo [30, 40–42]. To verify the self-assembly property of ELK16, two different fusions, GFP–Mxe–ELK16 and GFP–Mxe-His, were constructed, expressed in *E. coli* BL21(DE3) cells, and then analyzed with fluorescence confocal microscopy. As shown in Fig. 4, the fluorescent signal clearly localized the cellular distribution of GFP–Mxe–ELK16 (Fig. 4a), whereas the GFP–Mxe–His expressing cells displayed uniform green fluorescence throughout the cytoplasm (Fig. 4d). These data indicate that the ELK16 fusion could induce the formation of active protein aggregates in *E. coli* BL21(DE3) cells.

**Fig. 5:**
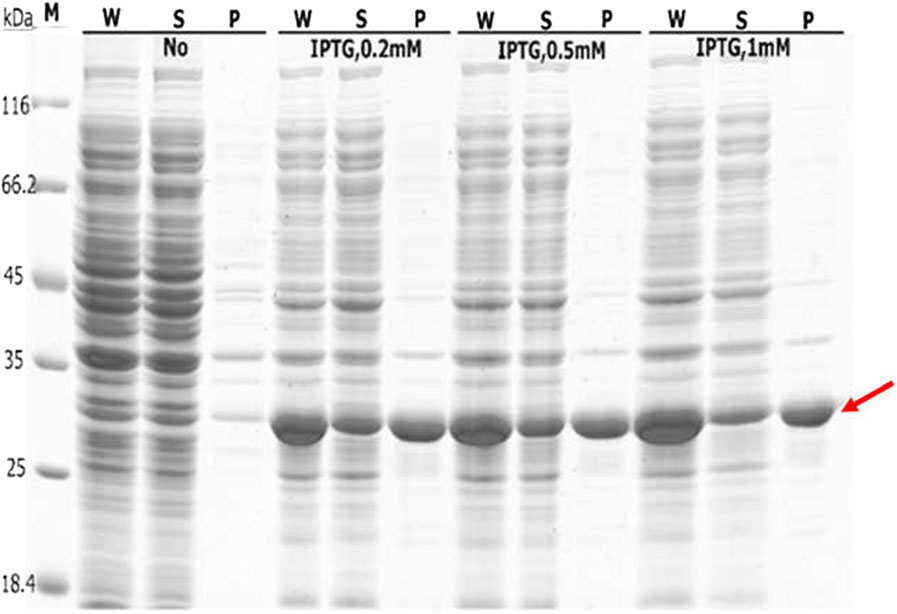
Expression of CeA–Mxe–ELK16 fusion in the SA-ELK16 system and its optimization. Cells producing the CeA–Mxe–ELK16 fusion were cultured at 16 °C for 24 h. Different concentrations of IPTG (0.2–1.0 mM) were tested to maximize expression. Positions corresponding to the CeA–Mxe–ELK16 fusion are marked with red arrows. NO, the cells were not induced with IPTG; W, whole-cell lysate; S, supernatant fractions; and P, insoluble pellets of induced cells

Based on the above results, ELK16 was chosen as the carrier protein fused to the CeA peptide (CeA–Mxe–ELK16) to induce the formation of active inclusion bodies in *E. coli*, achieving the highly efficient production of the CeA peptide. To prevent the formation of inclusion bodies, a lower temperature (16 °C) was selected as the induction temperature for 24 h, and different concentrations of IPTG (0.2, 0.5, and 1 mM) were tested. As shown in Fig. 5, a major band at approximately 30 kDa was observed, indicating that the CeA–Mxe–ELK16 fusion was successfully expressed. Approximately 50.23–78.16 μg/mg wet cell weight accumulated as insoluble aggregate and there was no intracellular self-cleavage of the fusion which reduced the toxicity to the host cells (Table 2). There was a slight increase in the expression of the CeA–Mxe–ELK16 fusion at a higher IPTG concentration (0.5 or 1 mM), and ultimately 1 mM IPTG was selected as the optimal concentration.

**Table 2:** Quantification of cecropin A peptides cleaved from the fusions in the three different schemes

Expression systems	MW (kDa)	Fusions yield^a^ (μg/mg wet cell pellet)	Peptide yield^b^ (μg/mg wet cell pellet)	Cleavage effciency^c^ (%)	Percent recovery^d^ (%)	Peptide purity^e^ (%)
CF system	4.6	─	0.93^f^	─	─	90.4
AT-HIS system	4.4	4.85	0.41	100	52.14	92.1
SA-ELK16 system	4.4	51.16	6.20	79.50	78.38	99.9

^a^Yield of fusion proteins in the form of aggregates or soluble fractions

^b^Yield of cecropin A peptides after intein-mediated cleavage in LB culture: 2.87 ± 0.62 mg/ml wet cell weight in the SA-ELK16 system and 1.48 ± 0.48 mg/ml wet cell weight in the AT-HIS system, estimated with the densitometric analysis software Gel-Pro Analyzer or BCA Protein Assay Kit

^c^Cleavage efficiency was calculated by dividing the amount of cleaved protein aggregate by the amount of total aggregate before cleavage

^d^Percentage recovery of cecropin A peptide was calculated by dividing the mass of cecropin A peptide after cleavage by the mass that could be theoretically obtained from the fusion protein

^e^Purity of CeA peptide was calculated with the BandScan software

^f^Yield of CeA peptide in the CF system was 0.93 μg/ml of reaction mixture

**Fig. 6:**
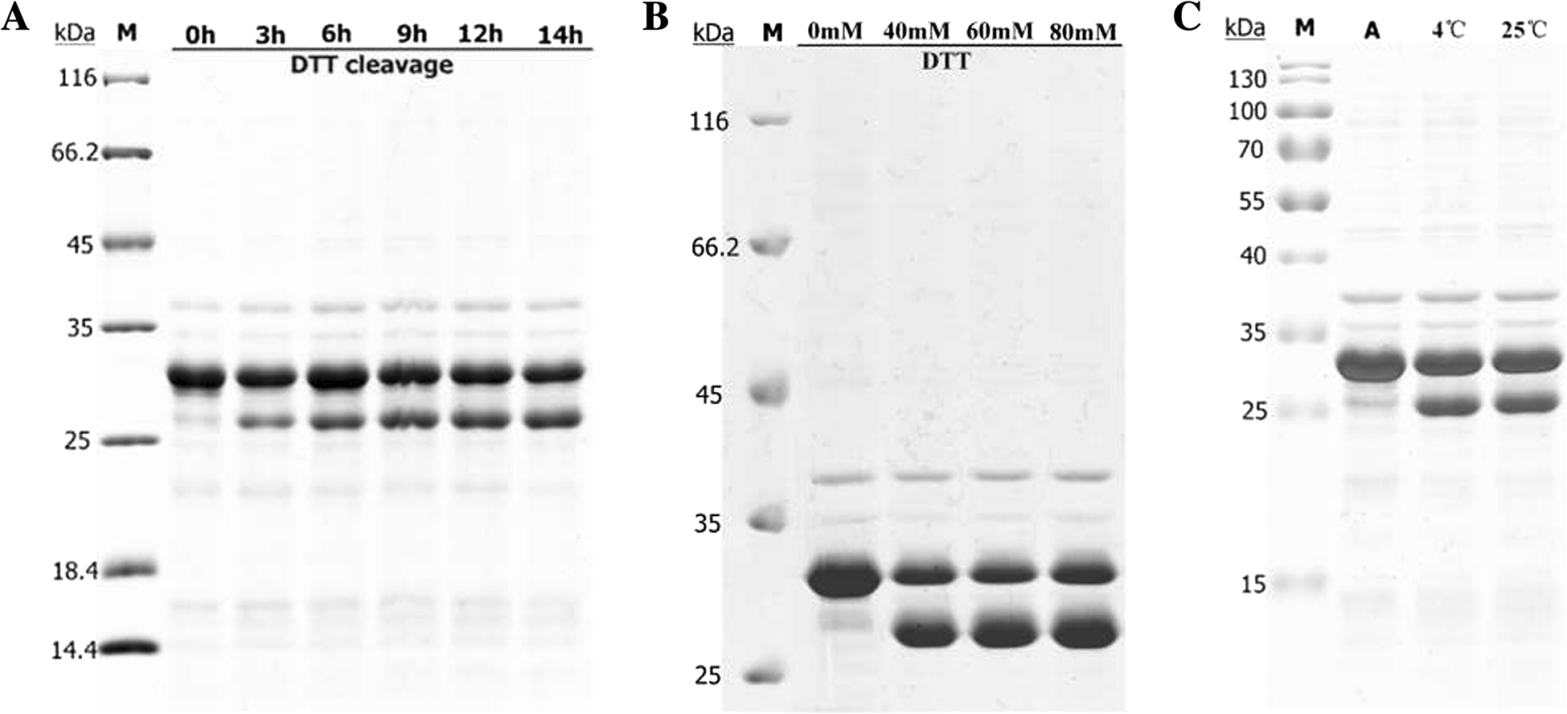
Combinatorial optimization analysis of *Mxe* GyrA intein cleavage activity in CeA–Mxe–ELK16 fusion. Different incubation times (**a**) and concentrations of DTT (40–80 mM) (**b**) were used to optimize cleavage activity. Different incubation temperatures (4 °C or 25 °C) were also tested (**c**). P, samples before cleavage with DTT; M, protein marker

**Fig. 7:**
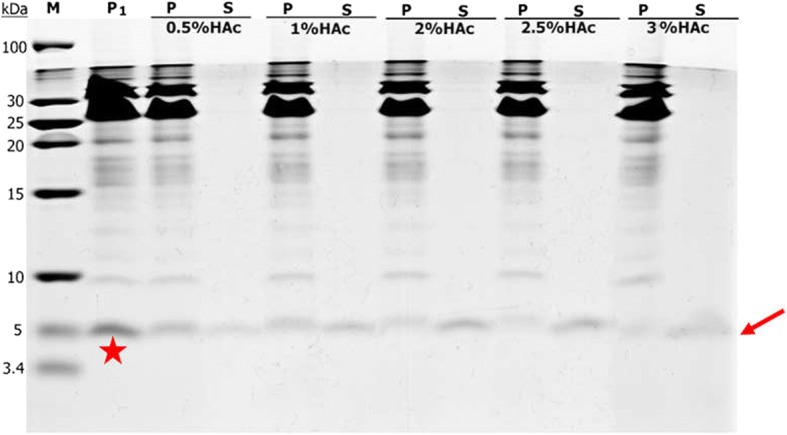
High-purity CeA peptide released with acetic acid treatment. Different concentrations of acetic acid (0.5–3% [*v*/v]) were used to release high-purity CeA peptide. Position corresponding to high-purity CeA peptide is marked with a red arrow. Red star indicates CeA peptide released from fusion protein. P_1_, insoluble fraction of the cleaved fusion protein; P, insoluble fraction after acetic acid treatment; S, soluble fraction after acetic acid treatment

To obtain more CeA peptide, the efficiency of *Mxe* GyrA intein cleavage was investigated and optimized. The CeA–Mxe–ELK16 aggregates were first subjected to cleavage with 40 mM DTT at 25 °C for 0–14 h. As shown in Fig. 6a, about 22.7% of the aggregates were cleaved after 3 h, and when the incubation time was extended to 12 h, nearly 46.7% of the fusion was cleaved, and this proportion remained constant with longer incubation time. DTT concentrations of 40–80 mM were also tested at 25 °C for 12 h, and 55–76.2% of the fusion aggregate was cleaved, indicating that 40 mM DTT was sufficient to induce the cleavage of the majority of CeA–Mxe–ELK16 aggregates (Fig. 6b and Table 2). Considering high temperature (25 °C) can reduce the stability and activity of the CeA peptide, different incubation temperatures (including 25 °C and 4 °C) were tested. As shown in Fig. 6c, incubation of the samples at 4 °C and 25 °C generated the same amounts of cleaved fusion aggregates. Based on these results, we performed all subsequent cleavage reactions with 40 mM DTT at 4 °C for 12 h. Then the cleaved samples were centrifuged at 12,000 g for 30 min at 4 °C. Unfortunately, the released CeA peptide remained in the insoluble fraction after centrifugation (band marked with a red star in Fig. 7-P_1_). According to recent studies, acetic acid treatment can denature and precipitate the majority of *E. coli* proteins [30, 43]. Moreover, the structure of CeA peptide is simple (only two α helices with no disulphide bond) and has a higher flexibility (it can fit its conformation to different circumstances) [44, 45]. Therefore, CeA peptide can resume a suitable structure in PBS buffer after acetic acid treatment. Based on this principle, different concentrations of acetic acid (0.5–3% [*v*/v]) were added to the cleaved samples to solubilize the CeA peptide. As can be seen in Fig. 7, CeA peptide of high purity (approximately 99.8%, Table 2) was successfully dissolved in the soluble fraction when the cleaved samples were incubated with acetic acid. Interestingly, increasing amounts of CeA peptide were dissolved in the soluble fraction as the increased amount addition of acetic acid added. Ultimately, the addition of 2.5% acetic acid was selected, because higher concentrations of acetic acid may affect the stability of CeA peptide. The dissolved CeA peptide was then concentrated and dialized in phosphate-buffered saline (PBS) for further study.

### Antibacterial properties of the bioproduced CeA peptide

**Fig. 8:**
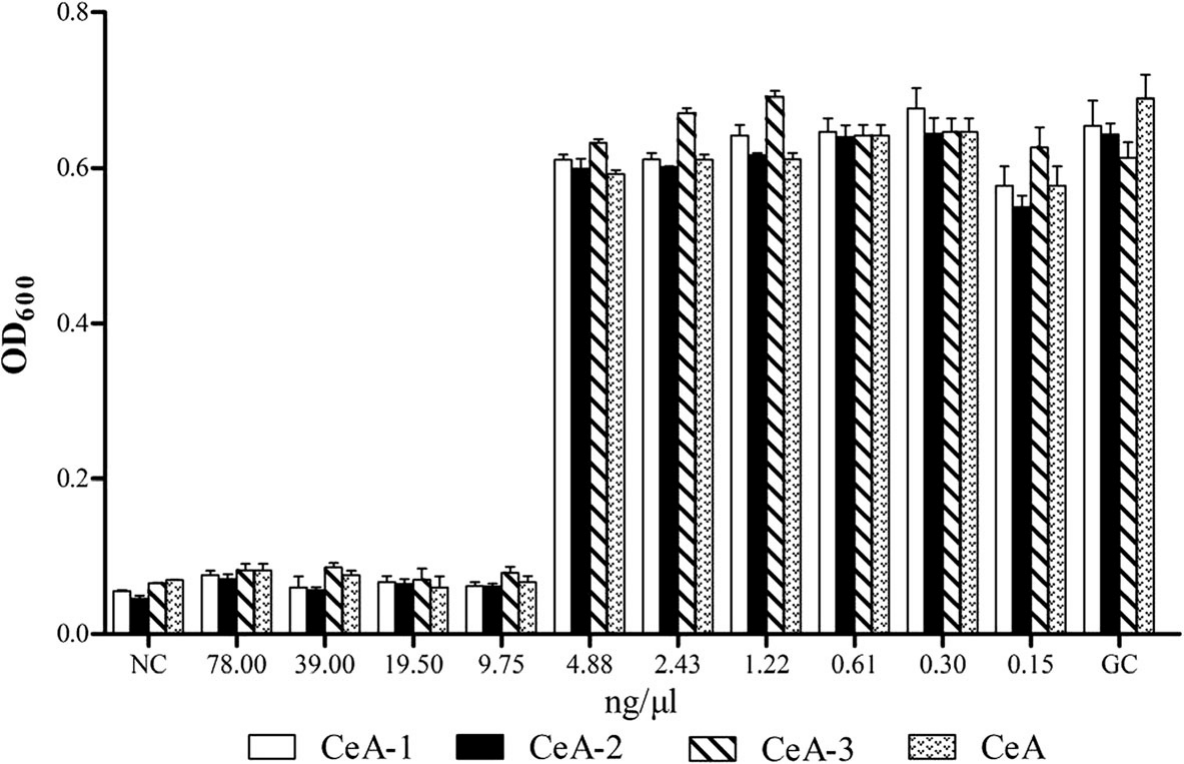
MIC assay against *E. coli* of CeA peptides produced in three different expression systems. Growth inhibition of *E. coli* ATCC 25922 is shown in the presence of various concentrations of CeA peptide produced in the CF system (white rectangle: CeA-1), AT-HIS system (black rectangle: CeA-2), and SA-ELK16 system (rectangle filled with oblique lines: CeA-3). Chemically synthesized CeA peptide was also tested (rectangle filled with point: CeA). GC, growth control for *E. coli* ATCC 25922 in the absence of CeA. NC, sterilized Müller–Hinton broth used as the normal control in this experiment. Experiments were performed in triplicate, and standard deviations (error bars) are shown

The in vitro antimicrobial activity against *E. coli* ATCC 25922 of the CeA peptide bio-produced with the three different expression systems was determined as the minimal inhibitory concentrations (MICs) with the broth microdilution method [45]. The results are shown in Fig. 8. The MICs of the CeA peptides generated in the three systems against *E. coli* ATCC25922 were the same (9.75 ng/μl), and consistent with the MIC of the chemically synthesized CeA peptide against *E. coli* ATCC 25922. These results indicate that the CeA peptides produced by these three protein expression systems had the same antimicrobial activity as synthetic CeA, and that the N-terminal additional of histidine to the CeA peptide in the CF system had no negative effect on its antibacterial activity.

## Discussion

In this study, we investigated three different protein expression systems for the production of the CeA peptide: the AT-HIS system, CF system, and SA-ELK16 system. By comparing the results, we developed a cost-effective and efficient protein expression system for high-purity CeA peptide.

In the AT-HIS system, two rounds of nickel ion affinity chromatography were required which was both expensive and time-consuming (approximately 5 days, Table 1). More importantly, a large amount of CeA peptide was lost in the second round of Ni^2+^ affinity chromatography, which significantly limited the large-scale production of CeA peptide. Finally, CeA peptide with 92.1% purity of was obtained at a yield of 0.41 μg/mg wet cell pellet, indicating that AT-HIS system is not an ideal system for the production of the CeA peptide. Due to its open nature and membrane-free, cell free protein expression system was considered the most suitable platform for expression of toxic proteins in recent years [46]. We tested the efficacy of the CF expression system for producing the CeA peptide. Table 1 shows that about 0.93 μg/ml reaction mixture of the CeA peptide was eventually collected, with 90.4% purity. When the CF and AT-HIS systems were compared, the CF system performed better than the AT-HIS system in that the final yield of CeA peptide was 2-fold higher than with the AT-HIS system. However, this yield is relatively low compared with the production of other AMPs, and falls far short of the yields required for large scale production [47, 48]. Moreover, the purity of the CeA peptide produced by these two expression systems was less than 95% and is inappropriate for further research. Most importantly, several cumbersome and expensive procedures like nickel affinity chromatography are required, which are not practicable for the large-scale production of the CeA peptide. Therefore, there is an urgent need to investigate and explore a high and efficient system for the production of CeA peptide.

To achieve a highly efficient low-cost production process for high-purity (> 98%) CeA peptide, the small self-assembling protein ELK16 was attempted in this study. In SA-ELK16 system, the recovery of CeA peptide was estimated to be 78.38%, higher than that in AT-HIS system (52.14%, Table 2). Finally, highly pure (~ 99.8%) CeA peptide was obtained after the cleavage of *Mxe* GyrA intein (Table 1), with a yield of 6.20 μg/mg wet cell pellet, which was 15-fold higher than the yield of the AT-HIS system and 6.7-fold higher than that of the CF protein expression system (Table 2). These results indicated that SA-ELK16 system was more efficient than AT-HIS system and CF system for production of CeA peptide. Furthermore, the CeA peptide was produced rapidly (2 days) with simple centrifugation and acetic acid treatment, eliminating expensive and tedious procedures like nickel affinity chromatography or high-performance liquid chromatography, dramatically reducing the production cost (Table 1). These factors make the SA-ELK16 system an ideal candidate for the laboratory scale production of the CeA peptide.

However, in SA-ELK16 system, much effort is still needed for the industrial production of CeA peptide. Such as the cleavage of CeA–Mxe–ELK16 fusion was induced by DTT in this study which was expensive at industry scale. Some other self-cleavage inteins like Ssp dnaB and sortase, which cleavage reactions were activated by pH and calcium ion, can be used to reduce the costs at industry production of CeA peptide [49–51]. Besides, the downstream process like dialysis also need to be further optimized to meet the needs of industrial production of CeA peptide.

## Conclusions

Cecropin A is a natural linear cationic AMP, with rapid and potent activity against a broad spectrum of pathogens, including bacteria, fungi, viruses, and neoplastic cells. Therefore, it has great potential utility in various biotechnological applications [2, 19, 22]. However, the exploitation of the CeA peptide is seriously limited by the low yields and expensive purification costs of its production. Here, we report a fast, economical and efficient expression system, the SA-ELK16 system, for the laboratory scale production of high-purity CeA peptide, which circumvents the difficulties encountered by ordinary recombinant methods or expensive chemical syntheses. It completely avoids the use of affinity resins and greatly reduces the cost and time required, providing a novel platform for laboratory scale production of the CeA peptide. This study lays a solid foundation for further research into the CeA peptide.

## Methods

### Strains and materials

Competent *E. coli* DH5α and BL21(DE3) cells were purchased from Tiangen Biotech (Beijing, China). Oligonucleotides for gene manipulation were synthesized by Invitrogen (Shanghai, China) or Tianyi Huiyuan Gene Technology (Guangzhou, China). The restriction endonuclease *Dpn*I used for restriction-free cloning (RF cloning) was obtained from Fermentas Thermo Scientific (Glen Burnie, MD, USA). The Hi Trap 5 mL pre-packed column used for protein purification was purchased from Qiagen (Dusseldorf, Germany). All chemical reagents used in this study were of analytical grade.

### Construction of recombinant plasmids

All three recombinant expression vectors used in the study were constructed with RF cloning. Like fusion PCR cloning, RF cloning uses the appropriate DNA fragment as a megaprimer for the linear amplification of the vector to introduce a foreign DNA into the plasmid at a predetermined position [38]. CeA peptide, *Mxe* GyrA intein, and ELK16 were synthesized with the appropriate DNA sequences by codon-optimized expression in *E. coli* BL21(DE3) by Sangon Biotech (Shanghai, China). The DNA fragments encoding the CeA peptide, *Mxe* GyrA intein, and ELK16 were cloned into the vector pET30a with RF cloning to construct pET30a–AM–ELK16. To construct pET21a–AM–His, primers AMH-F and AMH-R (see Additional file 2) were used to amplify the AM sequence encoding both the CeA peptide and the *Mxe* GyrA intein from plasmid pET30a-AM–ELK16. The amplified DNA fragment was then integrated into the pET21a vector with RF cloning. Plasmid pET21–His_6_–AMP was obtained by amplifying the CeA gene from pET30a–AM–ELK16 and inserting it into pET21a. Plasmid pET21–AMP–His_6_ was constructed in the same way as pET21–His_6_–AMP. All the primers used in this work are listed in Additional file 2.

### Expression of fusion proteins in three different systems

In the SA-ELK16 and AT-HIS systems, Luria–Bertani (LB) medium containing 50 μg/ml kanamycin or 100 μg/ml ampicillin was inoculated with *E. coli* BL21(DE3) cells carrying pET30a–AM–ELK16 or pET21a–AM–His, respectively, and incubated at 37 °C. Different concentrations of IPTG (0.1–1 mM) were added to initiate protein expression when the cell density (OD_600_) reached 0.6–08. The cultures were then incubated under different conditions (5 h/37 °C, 12 h/25 °C, or 24 h/16 °C) to optimize protein expression. The cells were harvested by centrifugation at 7500×g for 15 min and the pellets were stored at − 80 °C for further analysis. In the CF system, pET21–His_6_–AMP and its derivative were expressed with a previously described method [52]. The reaction solution, containing 60 μl of reaction mixture (RM) and 900 μl of feeding mixture (FM), was added to the wells of standard 24-well microplates. To optimize the Mg^2+^ concentration, regenerated cellulose membranes with a molecular-weight cut-off of 14 kDa were used. The reactions were performed in the continuous-exchange cell-free configuration and incubated for 12–16 h at 30 °C with shaking (180 rpm).

### Intein-mediated cleavage and peptide purification

In the CF system, the soluble fractions were harvested by centrifuging the reaction mixture at 12,000 g for 10 min. Ten-fold volumes of cold acetone were added to the supernatants to precipitate the protein [47]. The pellets were air dried, dissolved in 1 × loading buffer (20 mM Tris–HCl, 3% [*w*/*v*] glycerol, 1.5% [w/v] SDS, 5% [w/v] β-mercaptoethanol, 0.02% [w/v] bromophenol blue, pH 8.5), and denatured by heating at 95 °C for 10 min. The samples were separated and analyzed with 16% tricine SDS-PAGE. The CeA fusion produced with the CF system was purified with nickel ion affinity chromatography, as previously reported [39].

The harvested cell pellets containing the CeA–Mxe–ELK16 or CeA–Mxe–His fusions were resuspended to 10 OD_600_ units/ml of culture with lysis buffer (20 mM Tris-HCl, 500 mM NaCl, 1 mM EDTA, pH 8.5) and the suspended cells were lysed with a high-pressure homogenizer (Constant Systems Limited, United Kingdom). The CeA–Mxe–ELK16 aggregates were separated by centrifugation at 12,000 g for 30 min at 4 °C. The soluble fractions containing the CeA–Mxe–His fusions were isolated from the aggregates by centrifugation at 12,000 g for 30 min at 4 °C, and then purified with nickel ion affinity chromatography, as described above. For *Mxe* GyrA intein mediate cleavage, the CeA–Mxe–ELK16 aggregates or purified CeA–Mxe–His fusions were resuspended in cleavage buffer (20 mM Tris-HCl, 500 mM NaCl, 1 mM EDTA, pH 8.5) containing different concentrations of DTT (40–80 mM). The cleavage reaction was assessed by incubating the samples under different conditions (4 °C or 25 °C for 0–14 h) to optimize the efficiency of cleavage. The quantity of protein was determined with a BCA Protein Assay Kit (Sangon) or the Gel-Pro Analyzer software (Media Cybernetics, Houston, USA), using aprotinin as the standard and adjusting for the loading volume.

### Acetic acid treatment for high-purity CeA peptide in the SA-ELK16 system

To obtain high-purity (≥ 98%) CeA peptide, different proportions of acetic acid (0.5–3%) were added to the suspensions of the cleaved CeA–Mxe–ELK16 fusion and incubated at room temperature for 10 min. The supernatant containing high-purity CeA peptide was separated by centrifugation at 12,000 g for 30 min at 4 °C. The soluble and insoluble fractions were analyzed with tricine SDS-PAGE.

### Fluorescence confocal microscopy

*Escherichia coli* BL21(DE3) cells carrying the pET30–GFP–Mxe–ELK16 or pET21–GFP–Mxe–His plasmid were cultured and the expression of the fusion was induced by the addition of 0.2 mM IPTG. After expression, 200 μl of cells were harvested by centrifugation at 7000 g at 4 °C for 5 min, resuspended in sterile PBS (1% *w*/*v*), spotted onto a slide, air dried, and visualized with a 63× phase-contrast objective on a Zeiss LSM 710 microscope (Germany).

### MIC assay

The MICs of the CeA peptides produced in the three different expression systems against *E. coli* ATCC 25922 were assessed with the broth microdilution method, as previously reported [45], and compared with that of a synthetic CeA peptide. For the antimicrobial assays, overnight cultures of *E. coli* ATCC 25922 cells were diluted with fresh Müller–Hinton broth (MHB) to an OD_625_ of 0.08–0.13 (0.5 McFarland standards), and further diluted with fresh MHB (1:100) to a final concentration of approximately 10^7^ colony-forming units (CFU) per ml. The bio-produced CeA peptides were serially diluted in MHB medium and 50 μl aliquots of the diluted CeA peptides were added to 96-well plates. The standardized bacterial suspension (50 μl) was then added to each well. Growth controls contained no peptide antimicrobial, and sterility controls contained only broth. The plates were incubated at 37 °C for 16–18 h. The OD_600_ values were monitored with a microplate reader and the lowest concentration of peptide at which the growth of *E. coli* ATCC 25922 was inhibited was determined as the MIC.

## Additional files

Additional file 1: Purification of CeA-Mxe-His fusions in AT-HIS system. (DOCX 181 kb)

Additional file 2: Primers used in this study. (DOCX 145 kb)

## Abbreviations

CeA: Cecropin A
*E.coli*: *Escherichia coli*
FM: feeding mixture
IPTG: Isopropyl β-D-Thiogalactoside
LB: Luria-Bertani
MHB: Müller–Hinton broth
*Mxe*: Mxe GyrA intein
RF cloning: Restriction Free cloning
RM: reaction mixture

## References

[1] Schmitt P, Mercado L, Díaz M, Guzmán F, Arenas G, Marshall SH (2008). Characterization and functional recovery of a novel antimicrobial peptide (CEC dir–CEC ret) from inclusion bodies after expression in Escherichia coli. Peptides.

[2] DeLucca AJ, Bland JM, Jacks TJ, Grimm C, Cleveland TE, Walsh TJ (1997). Fungicidal activity of cecropin A. Antimicrob Agents Chemother.

[3] Cavallarin L, Andreu D, San Segundo B (1998). Cecropin A—derived peptides are potent inhibitors of fungal plant pathogens. Mol Plant-Microbe Interact.

[4] Andreu D, Merrifield R, Steiner H, Boman H (1983). Solid-phase synthesis of cecropin A and related peptides. Proc Natl Acad Sci.

[5] Silvestro L, Gupta K, Weiser JN, Axelsen PH (1997). The concentration-dependent membrane activity of cecropin A. Biochemistry.

[6] Zhang J, Movahedi A, Wang X, Wu X, Yin T, Zhuge Q (2015). Molecular structure, chemical synthesis, and antibacterial activity of ABP-dHC-cecropin A from drury (Hyphantria cunea). Peptides.

[7] Thinkhamrop J, Hofmeyr GJ, Adetoro O, Lumbiganon P (2002). Prophylactic antibiotic administration in pregnancy to prevent infectious morbidity and mortality. Cochrane Database Syst Rev.

[8] Gauger PC, Loving CL, Khurana S, Lorusso A, Perez DR, Kehrli ME, Roth JA, Golding H, Vincent AL (2014). Live attenuated influenza a virus vaccine protects against a (H1N1) pdm09 heterologous challenge without vaccine associated enhanced respiratory disease. Virology.

[9] Paudel S, Park J, Jang H, Hyun B, Yang D, Shin H (2014). Evaluation of antibody response of killed and live vaccines against porcine epidemic diarrhea virus in a field study. Vet Q.

[10] Wu R, Wang Q, Zheng Z, Zhao L, Shang Y, Wei X, Liao X, Zhang R (2014). Design, characterization and expression of a novel hybrid peptides melittin (1–13)-LL37 (17–30). Mol Biol Rep.

[11] Zhang J, Li J, Movahedi A, Sang M, Xu C, Xu J, Wei Z, Yin T, Zhuge Q (2015). A novel inclusion complex (β-CD/ABP-dHC-cecropin A) with antibiotic propertiess for use as an anti-Agrobacterium additive in transgenic poplar rooting medium. Enzym Microb Technol.

[12] Efimova SS, Schagina LV, Ostroumova OS (2014). Channel-forming activity of cecropins in lipid bilayers: effect of agents modifying the membrane dipole potential. Langmuir.

[13] Yi H-Y, Chowdhury M, Huang Y-D, Yu X-Q (2014). Insect antimicrobial peptides and their applications. Appl Microbiol Biotechnol.

[14] Kylsten P, Samakovlis C, Hultmark D (1990). The cecropin locus in Drosophila; a compact gene cluster involved in the response to infection. EMBO J.

[15] X-m L, X-b J, J-y Z, H-f M, Ma Y, F-j C, Wang Y, Li X-b (2010). Expression of the antimicrobial peptide cecropin fused with human lysozyme in Escherichia coli. Appl Microbiol Biotechnol.

[16] Zhou X, Li X, Wang X, Jin X, Shi D, Wang J, Bi D. Cecropin B represses CYP3A29 expression through activation of the TLR2/4-NF-κB/PXR signaling pathway. Sci Rep. 2016;6. 10.1038/srep27876. 10.1038/srep27876 PMC4906279 27296244

[17] Bechinger B (1997). Structure and functions of channel-forming peptides: magainins, cecropins, melittin and alamethicin. J Membr Biol.

[18] Boman H, Boman IA, Andreu D, Li Z-Q, Merrifield R, Schlenstedt G, Zimmermann R (1989). Chemical synthesis and enzymic processing of precursor forms of cecropins A and B. J Biol Chem.

[19] Hancock RE, Lehrer R (1998). Cationic peptides: a new source of antibiotics. Trends Biotechnol.

[20] Gordon YJ, Romanowski EG, McDermott AM (2005). A review of antimicrobial peptides and their therapeutic potential as anti-infective drugs. Curr Eye Res.

[21] Xu X, Jin F, Yu X, Ji S, Wang J, Cheng H, Wang C, Zhang W (2007). Expression and purification of a recombinant antibacterial peptide, cecropin, from Escherichia coli. Protein Expr Purif.

[22] Andersons D, Engström Å, Josephson S, Hansson L, Steiner H (1991). Biologically active and amidated cecropin produced in a baculovirus expression system from a fusion construct containing the antibody-binding part of protein a. Biochem J.

[23] Yang K, Su Y, Li J, Sun J, Yang Y (2012). Expression and purification of the antimicrobial peptide cecropin AD by fusion with cationic elastin-like polypeptides. Protein Expr Purif.

[24] Téllez GA, Castaño-Osorio JC (2014). Expression and purification of an active cecropin-like recombinant protein against multidrug resistance Escherichia coli. Protein Expr Purif.

[25] Ji S, Li W, Baloch AR, Wang M, Li H, Cao B, Zhang H. Efficient biosynthesis of a Cecropin A-melittin mutant in Bacillus subtilis WB700. Sci Rep. 2017;7. 10.1038/srep40587. 10.1038/srep40587 PMC5223193 28071737

[26] F-l J, X-x X, X-q Y, S-x R (2009). Expression and characterization of antimicrobial peptide CecropinAD in the methylotrophic yeast Pichia pastoris. Process Biochem.

[27] Shen A, Lupardus PJ, Morell M, Ponder EL, Sadaghiani AM, Garcia KC, Bogyo M (2009). Simplified, enhanced protein purification using an inducible, autoprocessing enzyme tag. PLoS One.

[28] Liang Y, Wang J-X, X-f Z, Du X-J, Xue J-F (2006). Molecular cloning and characterization of cecropin from the housefly (Musca domestica), and its expression in Escherichia coli. Dev Compar Immunol.

[29] Xia L, Liu Z, Ma J, Sun S, Yang J, Zhang F (2013). Expression, purification and characterization of cecropin antibacterial peptide from Bombyx mori in Saccharomyces cerevisiae. Protein Expr Purif.

[30] Zhou B, Xing L, Wu W, Zhang X-E, Lin Z (2012). Small surfactant-like peptides can drive soluble proteins into active aggregates. Microb Cell Factories.

[31] Xu W, Zhao Q, Xing L, Lin Z. Recombinant production of influenza hemagglutinin and HIV-1 GP120 antigenic peptides using a cleavable self-aggregating tag. Sci Rep. 2016;6. 10.1038/srep35430. 10.1038/srep35430 PMC5093863 27808126

[32] Da Costa JP, Cova M, Ferreira R, Vitorino R (2015). Antimicrobial peptides: an alternative for innovative medicines?. Appl Microbiol Biotechnol.

[33] Satakarni M, Curtis R (2011). Production of recombinant peptides as fusions with SUMO. Protein Expr Purif.

[34] Ma Y, Yu J, Lin J, Wu S, Li S, Wang J (2016). High efficient expression, purification, and functional characterization of native human epidermal growth factor in Escherichia coli. Biomed Res Int.

[35] Jia C, Wang S, Chen Y, Xiao X, Su Z, Zheng Q (2017). Improving Soluble Expression of Curcin-Transferrin Receptor-binding Peptide Fusion Protein with Glutathione Transferase-Small Ubiquitin-related Modifier System. J Pharm Biomed Sci.

[36] Chen Y, Tan S, Yang F, Chen Z, Wu Z, Huang J (2017). Soluble expression and purification of a functional harpin protein in Escherichia coli. Process Biochem.

[37] Marshall CJ, Grosskopf VA, Moehling TJ, Tillotson BJ, Wiepz GJ, Abbott NL, Raines RT, Shusta EV (2014). An evolved Mxe GyrA intein for enhanced production of fusion proteins. ACS Chem Biol.

[38] Ent FVD, Löwe J (2006). RF cloning: a restriction-free method for inserting target genes into plasmids. J Biochem Biophys Methods.

[39] Wang M, Huang M, Gu H, Li S, Ma Y, Wang J (2017). Mutational analysis to identify the residues essential for the acetyltransferase activity of GlmU in Bacillus subtilis. RSC Adv.

[40] Ge X, Yang DS, Trabbic-Carlson K, Kim B, Chilkoti A, Filipe CD (2005). Self-cleavable stimulus responsive tags for protein purification without chromatography. J Am Chem Soc.

[41] Vázquez E, Villaverde A (2010). Engineering building blocks for self-assembling protein nanoparticles. Microb Cell Factories,9,1(2010-12-30).

[42] Lin Zhanglin, Zhou Bihong, Wu Wei, Xing Lei, Zhao Qing (2013). Self-assembling amphipathic alpha-helical peptides induce the formation of active protein aggregates in vivo. Faraday Discussions.

[43] Pane Katia, Durante Lorenzo, Pizzo Elio, Varcamonti Mario, Zanfardino Anna, Sgambati Valeria, Di Maro Antimo, Carpentieri Andrea, Izzo Viviana, Di Donato Alberto, Cafaro Valeria, Notomista Eugenio (2016). Rational Design of a Carrier Protein for the Production of Recombinant Toxic Peptides in Escherichia coli. PLOS ONE.

[44] Holak TA, Engström A, Kraulis PJ, Lindeberg G, Bennich H, Jones TA, Gronenborn AM, Clore GM (1988). The solution conformation of the antibacterial peptide cecropin A: a nuclear magnetic resonance and dynamical simulated annealing study. Biochemistry.

[45] Lee E, Shin A, Kim Y (2015). Anti-inflammatory activities of cecropin A and its mechanism of action. Arch Insect Biochem Physiol.

[46] Wiegand I, Hilpert K, Hancock REW (2008). Agar and broth dilution methods to determine the minimal inhibitory concentration (MIC) of antimicrobial substances. Nat Protoc.

[47] Ma Y, Ghoshdastider U, Wang J, Ye W, Dötsch V, Filipek S, Bernhard F, Wang X (2012). Cell-free expression of human glucosamine 6-phosphate N-acetyltransferase (HsGNA1) for inhibitor screening. Protein Expr Purif.

[48] Rao X, Li S, Jc JX, Hu X, Huang J, Chen Z, Zhu J, Hu F (2004). A novel carrier molecule for high-level expression of peptide antibiotics in Escherichia coli. Protein Exp Purification.

[49] Feng X, Liu C, Guo J, Song X, Li J, Xu W, Li Z (2012). Recombinant expression, purification, and antimicrobial activity of a novel hybrid antimicrobial peptide LFT33. Appl Microbiol Biotechnol.

[50] Vieweg S, Ansaloni A, Wang ZM, Warner JB, Lashuel HA (2016). An Intein-based strategy for the production of tag-free huntingtin exon 1 proteins enables new insights into the Polyglutamine dependence of Httex1 aggregation and fibril formation. J Biol Chem.

[51] Hay ID, Du J, Reyes PR, Rehm BHA (2015). In vivo polyester immobilized sortase for tagless protein purification. Microb Cell Factories.

[52] Ma Y, Muench D, Schneider T, Sahl H-G, Bouhss A, Ghoshdastider U, Wang J, Doetsch V, Wang X, Bernhard F (2011). Preparative scale cell-free production and quality optimization of MraY homologues in different expression modes. J Biol Chem.

