# Supplementary material for: Rapid and efficient production of cecropin A antibacterial peptide in Escherichia coli by fusion with a self-aggregating protein
Source: BMC Biotechnol. 2018 Oct 5;18:62. doi: 10.1186/s12896-018-0473-7 (PMC6173929; doi:10.1186/s12896-018-0473-7)
Supplement: Supplementary file 1 — Purification of CeA-Mxe-His fusions in AT-HIS system. (DOCX 181 kb) [file 12896_2018_473_MOESM1_ESM.docx]

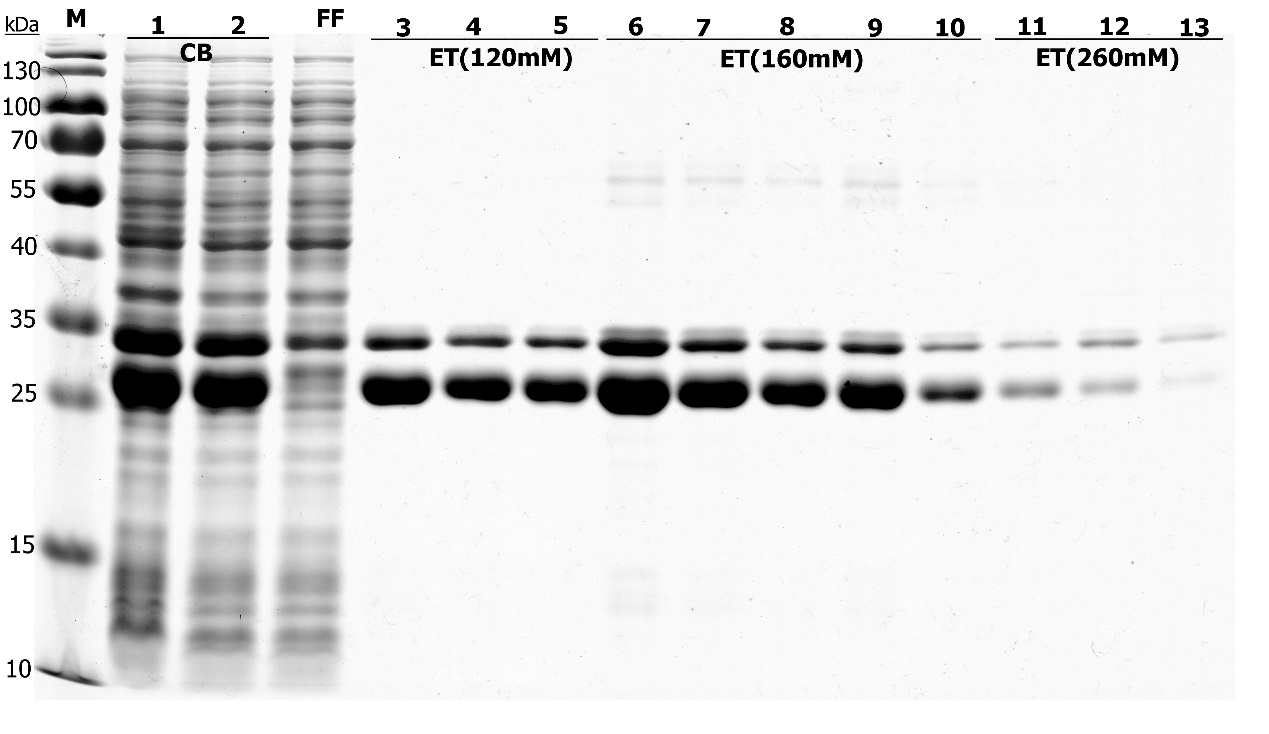


**Figure. S1 Purification of CeA-Mxe-His fusions in AT-HIS system**

Cells harboring CeA-Mxe-His fusions were disrupted by high-pressure cell crusher and then purified by nickel ion affinity chromatography. CB (1,2): the supernatant before loading on the column; “ET” and “FF” indicate elution and fast flow fractions; M represents the protein marker.
