# Supplementary figures and images for: Rapid and efficient production of cecropin A antibacterial peptide in Escherichia coli by fusion with a self-aggregating protein
Source: BMC Biotechnol. 2018 Oct 5;18:62. doi: 10.1186/s12896-018-0473-7 (PMC6173929; doi:10.1186/s12896-018-0473-7)

**Table S1 Primers used in this study**


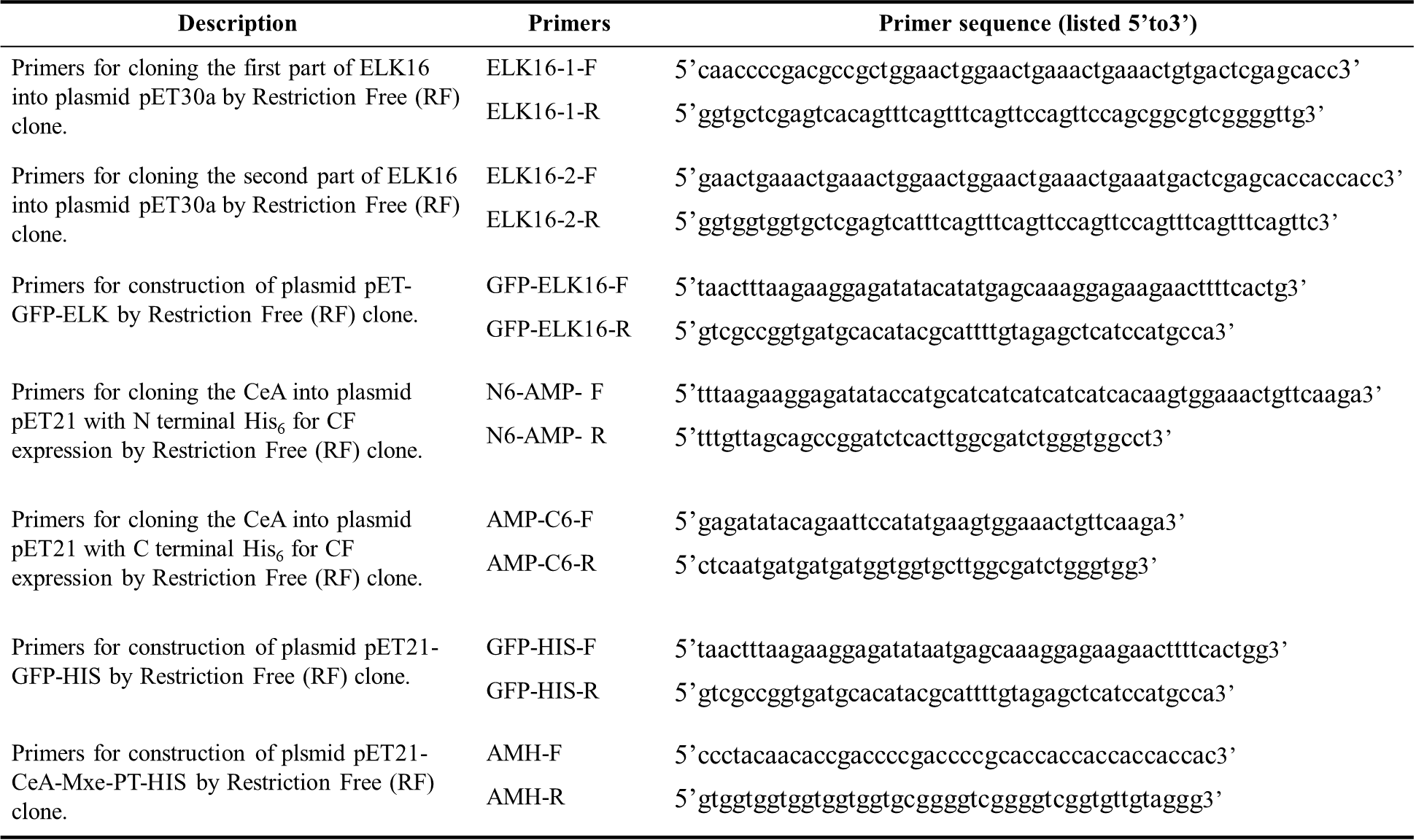

Supplement: Supplementary file 2 — Primers used in this study. (DOCX 145 kb) [file 12896_2018_473_MOESM2_ESM.docx]
